# Sachet water in Ghana: A spatiotemporal analysis of the recent upward trend in consumption and its relationship with changing household characteristics, 2010–2017

**DOI:** 10.1371/journal.pone.0265167

**Published:** 2022-05-26

**Authors:** Simon Moulds, Anson C. H. Chan, Jacob D. Tetteh, Honor Bixby, George Owusu, Samuel Agyei-Mensah, Majid Ezzati, Wouter Buytaert, Michael R. Templeton

**Affiliations:** 1 School of Geography and the Environment, University of Oxford, Oxford, United Kingdom; 2 Department of Civil and Environmental Engineering, Imperial College London, London, United Kingdom; 3 Department of Geography and Resource Development, University of Ghana, Accra, Ghana; 4 Department of Epidemiology, Biostatistics and Occupational Health, McGill University, Montreal, Canada; 5 Institute of Statistical, Social and Economic Research, University of Ghana, Accra, Ghana; 6 MRC Centre for Environment and Health, School of Public Health, Imperial College London, London, United Kingdom; 7 Abdul Latif Jameel Institute for Disease and Emergency Analytics, Imperial College London, London, United Kingdom; 8 Regional Institute for Population Studies, University of Ghana, Accra, Ghana; University of Texas at Arlington, UNITED STATES

## Abstract

The consumption of packaged water in Ghana has grown significantly in recent years. By 2017, “sachet water”—machine-sealed 500ml plastic bags of drinking water—was consumed by 33% of Ghanaian households. Reliance on sachet water has previously been associated with the urban poor, yet recent evidence suggests a customer base which crosses socioeconomic lines. Here, we conduct a repeated cross-sectional analysis of three nationally representative datasets to examine the changing demography of sachet water consumers between 2010 and 2017. Our results show that over the course of the study period sachet water has become a ubiquitous source of drinking water in Ghana, with relatively wealthy households notably increasing their consumption. In 2017, the majority of sachet water drinking households had access to another improved water source. The current rate and form of urbanisation, inadequate water governance, and an emphasis on cost recovery pose significant challenges for the expansion of the piped water supply network, leading us to conclude that sachet water will likely continue to be a prominent source of drinking water in Ghana for the foreseeable future. The main challenge for policymakers is to ensure that the growing sachet water market enhances rather than undermines Ghana’s efforts towards achieving universal and equitable access to clean drinking water and sanitation.

## Introduction

In many West African nations the rate of urbanisation has outpaced official efforts to expand the provision of safe drinking water [1]. Instead, many households have turned to the private sector to fulfil their drinking water demand [2]. Among the modes and mediums used by the private sector to supply water, the market for sachet water—machine-sealed 500ml plastic bags of drinking water—has risen dramatically over the last two decades in West African nations including Ghana and Nigeria [3]. As the previous ideal of a single water utility providing a consistent and universal service has come to be seen as increasingly unrealistic for many developing countries [4, 5], the potential of the private sector to expand the provision of safe drinking water to underserved populations has received significant attention in recent years [5]. However, there are concerns that the growing sachet water market, left unchecked, may undermine the principle of water as a public good and threaten the goal of universal access [5–7]. As policymakers grapple with these issues, there is a need to understand the role of sachet water in shaping recent trends in West Africa’s drinking water landscape. This study aims to contribute to the emerging discourse on sachet water consumption in West Africa by identifying spatiotemporal trends in drinking water preferences among households in Ghana—one of the largest markets for sachet water in the region—between 2010 and 2017.

The purchase of small volumes of drinking water has a long history in Ghana [5]. In the 1970s and 1980s it was common for Ghanaian street vendors to sell drinking water in cups scooped out of water storage tanks [1]. Increasing demand and hygiene concerns through the 1990s led to water being sold in plastic bags with the corners tied at the top, sometimes chilled by ice-blocks [1, 8]. Around the same time, Chinese machines which packaged filtered water into heat-sealed plastic sleeves started to be imported, creating sachet water as it is known today [3]. Sachet water—popularly known as “pure water” [5]—has the second-highest cost by volume after bottled water [9]. Nevertheless, its unit price of around 0.05 USD [9] remains within reach of those in absolute poverty [10]. The water source used to produce sachet water varies by location. In Accra, it is typically produced in parts of the city with a reliable connection to the piped supply network [1], while in peri-urban and rural areas manufacturers often rely on groundwater [1, 11]. Several studies have shown that sachet water exhibits low levels of contamination compared with other drinking water sources [9, 12, 13].

Vended water is often taken as a symptom of failure in the piped water supply networks [14]. Undoubtedly, the rapid growth of Ghana’s sachet water market has been fuelled by water resources mismanagement, water governance failures, and long-term neglect of water infrastructure [3, 15]. Ghana’s water supply infrastructure has not been strategically developed since independence in 1957 [16], with the result that spatial disparities in water access inherent to the colonial water system persist to this day [17]. The pressure on municipal water services has been exacerbated by rapid population growth and urbanisation, with Ghana’s urban population increasing from around 4 million in 1984 to more than 16 million in 2017 [18]. The failure to properly manage this process—albeit in the face of considerable political and economic challenges—has precluded a strategic approach to expanding the coverage of the piped supply network [19]. Meanwhile, the proliferation of unplanned urban settlements has challenged the role of the state in providing basic services to those living informally [20]. Given an unreliable and sometimes non-existent municipal water source, Ghanaian households have tended to diversify their water sources to ensure their drinking water demand is consistently met [9, 21]. Sachet water is appealing in this respect as it can be easily vended, transported, and stored before consumption as a primary or supplementary drinking water source, either inside or outside of the home [5].

Household survey data from Accra collected during 2009–2010 linked sachet consumption with the urban poor [10], with 50% of households from an informal settlement reporting sachet water as their primary source of drinking water. The survey data revealed that sachet drinking households were more likely to live in informal dwellings (e.g. huts, tents), use inferior bathing facilities (e.g. open spaces, rivers), cook with charcoal, and lack drainage infrastructure and access to electricity [10], leading to the conclusion that sachet water drinking households in urban Accra tended to be the “poorest of the poor” [10]. These findings were supported by a 2008 survey of women in informal settlements in Accra [22], which also found that sachet water consumers were generally younger with lower self-reported health. However, an opposing trend was observed in an informal settlement with reliable piped water supply in Ashaiman, a city in Greater Accra, where sachet water consumption was associated with proxies of higher disposable income [23]. Among the survey participants, more than half used sachet water as their primary drinking water source, with convenience and better perceived water quality cited as the main reasons for their choice. Of the respondents who did not drink sachet water most cited a lack of affordability as the main reason for their choice, with only a minority referring to a lack of piped water.

Ultimately, the dramatic increase in sachet water consumption in Ghana has been driven by the failure of municipal water supplies to reach large swathes of the population [10, 22]. Moreover, many of those with nominal access to the piped network report an unreliable service [10], and a perception of poor water quality [5]. The supposed convenience of piped water is further diminished for those who are obliged to use communal standpipes [10]. At the same time, it is clear that many consumers are attracted by the convenience and perceived water quality of sachet water [23]. While demand side factors have positioned sachet water as a highly appealing product, the rapid growth of the sachet water market over the last two decades would not have been possible without significant changes on the supply side which have enabled manufacturers to rapidly scale up production to meet demand, maintain a degree of control on quality standards, and resist periodic government threats to ban the industry [3].

As the aim of expanding the coverage of municipal piped supply networks comes to be seen as both unrealistic and perhaps outdated [24], it seems likely that sachet water will have a noticeable influence on Ghana’s progress towards the United Nations Sustainable Development Goal 6.1 (SDG 6.1), which aims towards “universal and equitable access to safe and affordable drinking water for all” by the year 2030 [25]. Some scholars have suggested that integrating packaged water into water delivery governance frameworks appears the best way to address remaining concerns around water quality and environmental sustainability [3]. While the capacity of the sachet water market to widen access to safe drinking water is clear, there are outstanding questions about the social justice implications of formally integrating sachet water into Ghana’s drinking water landscape. In particular, there is concern that such a move would legitimize the provision of water as a discrete private good, and undermine efforts towards universal to safe drinking water [5, 24]. In itself, increasing the regulatory burden on the sachet water sector may increase costs to consumers, while doing little to curb the proliferation of small-scale informal producers which are most susceptible to water quality issues [24]. At the same time, there are fears that focusing on sachet water as a solution to Ghana’s water supply deficit ignores problems related to the supply of non-drinking water [5].

Statistical reports indicate that sachet water consumption increased rapidly between 2010 and 2017 [26–28]. During the same period Ghana’s gross domestic product (GDP) [29] and human development index (HDI) [30] increased considerably. The continued rise in sachet water consumption in Ghana over the past decade raises important questions for Ghana’s water security. Do sachet water consumers still mostly belong to low-income households? Is sachet water consumption restricted to urban areas, or is it gaining market share in rural areas? Is there evidence that sachet water consumption is increasing outside of Accra? As the private sector grows, how does the state manage progress towards universal access to clean and affordable drinking water? Answering these questions is vital to inform sustainable and equitable water resource management policies towards universal access to clean drinking water and sanitation. To that end, we conduct a repeated cross-sectional analysis of three nationally representative datasets from 2010, 2013 and 2017 to gain an updated understanding of the changing demography of sachet water consumption in Ghana. The paper is structured as follows. In the next section we describe the three datasets we use in the study and the analyses we have carried out in order to answer the research questions outlined above. We present our results and discuss our findings in the context of previous studies on sachet water consumption and the tension between centralised and decentralised water supply systems. Lastly, we draw brief conclusions and outline future research priorities.

## Materials and methods

### Research design

Our study follows a quantitative research design consisting of descriptive and correlational elements. We first implemented a descriptive design to identify recent trends in the consumption of drinking water in Ghana. We then followed a correlational design to establish relationships between the observed trends in drinking water consumption and other aspects of socioeconomic development. At each time point we computed the global and local Moran’s I to identify the presence of spatial autocorrelation at the district level, and its variability in space. We performed a repeated measures correlation analysis to assess the relationship between sachet water consumption and multiple household characteristics consistently recorded over the three surveys, as well as subnational human development indices. The analysis was performed in the statistical programming language R (http://cran.r-project.org/).

### Input data

Our analysis draws on three national surveys comprising the 2010 Population and Housing Census [26] and the 2013 and 2017 Ghana Living Standards Survey (GLSS) [27, 28]. These datasets have a nationally representative sample and include information on primary drinking water sources as well as household characteristics relating to sanitation and access to technologies. Ghana’s 2010 decennial Population and Housing Census was the first study conducted by the Ghana Statistical Service (GSS) which recognised the “patchwork” of water sources consumed by Ghanaian households [2, 26]. A notable change in the 2010 census compared to previous censuses included inquiring about the household’s source of drinking and household (non-drinking) water. In addition, it was the first census of Ghana which included packaged water (divided between “bottled water” and “sachet water”) as possible answer choices for expressing drinking water preferences. The 2010 census was a nationwide study which enumerated every person in Ghana—irrespective of nationality—on the midnight of 26th September 2010 [26]. Here, we use the 10% sample of census records which are in the public domain, comprising a random sample of 10% of records from each enumeration area (*n* = 37, 642).

The GLSS studies are held by the GSS in the years between the decennial censuses. The most recent GLSS studies were conducted in 2013 (GLSS6) and 2017 (GLSS7). In comparison to the Population and Housing Census, which aims for a general overview of the population, the GLSS studies are specifically designed to measure the living conditions and wellbeing of Ghana’s residents, profiling participants to a greater depth than is possible in the main census. The GLSS studies do not sample Ghana’s entire population but instead rely on a smaller representative sample. Each household included in the survey is weighted according to the inverse of the probability of selection so that the results reflect Ghana’s entire population.

### Data harmonisation

**Fig 1:**
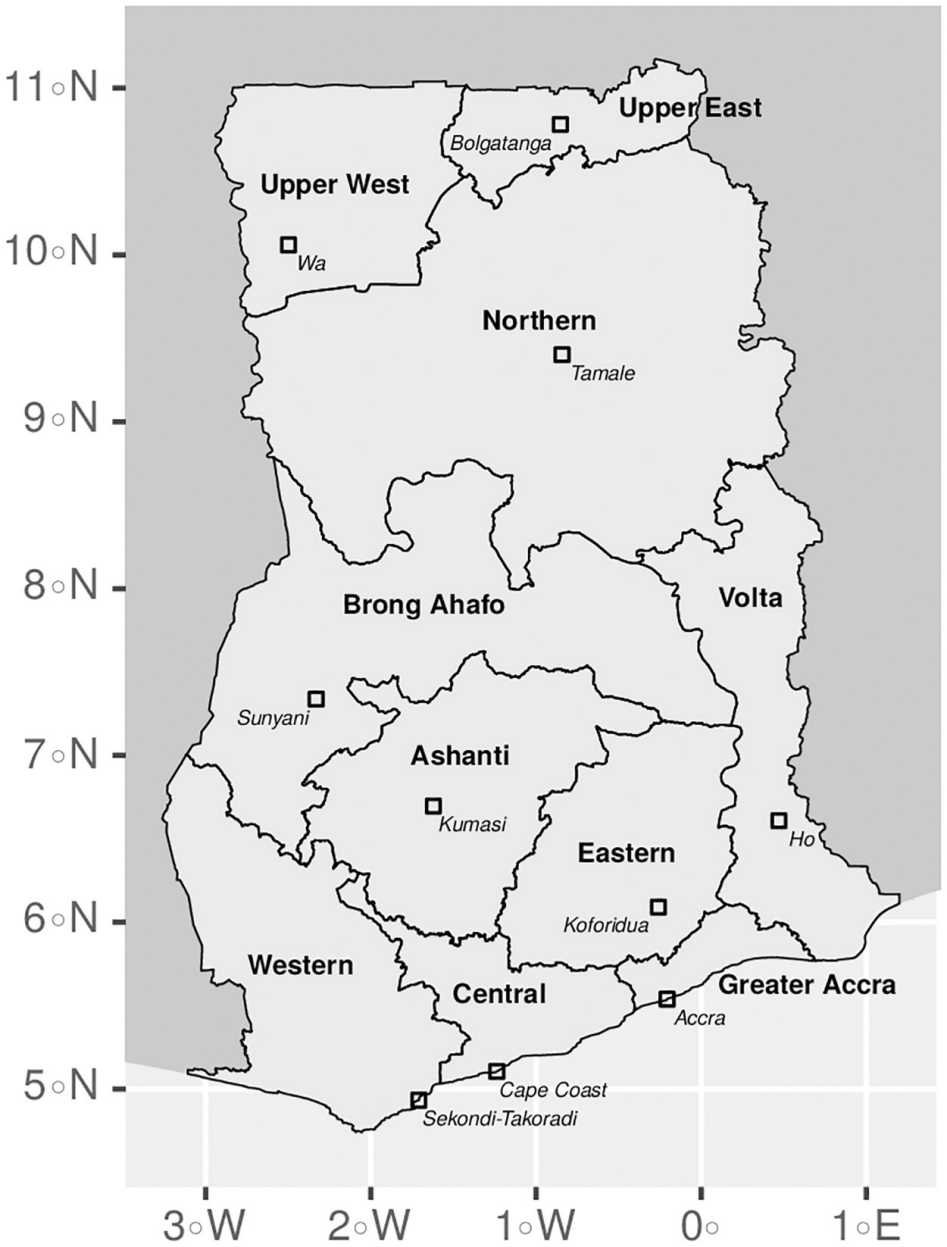
Administrative regions and regional capitals of Ghana. The regional boundaries shown are consistent with those in place during the study period (2010–2017), although the number of regions has subsequently increased to 16. Maps were created with data from the Humanitarian Data Exchange [33], which are made available under a CC-BY license [34].

Comparisons between the census and the GLSS datasets are possible because the questions asked in the GLSS studies are in general a superset of those asked in the census, albeit with minor wording differences in the questions and possible answers. Both the 2010 Population and Housing Census and the GLSS studies record the household’s primary source of drinking water rather than the preferences of individual household members, allowing a direct comparison between the results. During the analysis we applied sample weights to correct imbalances between the respective samples and the population as a whole. We first computed weights to account for missing data, by dividing the total number of households by the number of households who provided a response to each of the survey questions relevant to the analysis outlined here. Weights were computed at the district level. The 10% sample of the 2010 Population and Housing Census is assumed to be representative of the entire population, so we multiplied the weights accounting for missing data by a factor of 10 and applied these across the dataset. For the GLSS studies we computed missing data weights in the same way, and multiplied these by the precomputed sample weights which are provided with the dataset. To analyse spatial trends in the data we linked the datasets with spatial polygons of Ghana’s districts (*n* = 170) and regions (*n* = 10), which were obtained from GSS [31, 32] (Fig 1). In 2012, the number of administrative districts in Ghana was increased from 170 to 216 by dividing several existing districts. To allow for comparisons between the datasets we reprocessed the Population and Housing Census and GLSS7 data, which reference the post-2012 boundaries, to instead use pre-2012 classification. The number of administrative regions did not change during the study period, although we note that Ghana has subsequently been divided into 16 regions.

### Statistical analysis

#### Trends in drinking water preferences

To analyse trends in drinking water sources we reclassified the water sources used in the Population and Housing Census and GLSS surveys to the system used by the Joint Monitoring Program (JMP) of UNICEF/WHO, allowing us to distinguish between improved and unimproved water sources (Table 1). In general, improved sources include piped water and groundwater that is safely sourced, while unimproved sources comprise surface water that is unsafely sourced [35]. Since 2017 the JMP has treated sachet water and bottled water as improved sources in recognition of their generally superior quality compared with unimproved sources [35]. Prior to 2017 the JMP had taken a two-staged approach, with packaged water defined as an improved source only when the source of non-drinking water used by the household is also an improved source [36]. Here, we follow the post-2017 approach of the JMP in classifying sachet water as an improved source regardless of the household water source. However, to provide additional insight we distinguish between sachet water consuming households which use improved water for non-drinking purposes versus those which use unimproved water sources. We note that the JMP definitions have been supplemented by a “service ladder” which divides improved sources into safely managed, basic, and limited service levels [37]. Unfortunately the census data contains insufficient detail to classify water sources according to this framework.

**Table 1:** Correspondence between the JMP classification system and that used by the three surveys included in the present study.

JMP	Survey data
Improved: Piped water (private)	Pipe-borne (inside dwelling)
	Pipe-borne (outside dwelling)*
Improved: Piped water (public)	Public tab, standpipe
Improved: Groundwater	Borehole, pump, tubewell
	Protected well
Improved: Others	Protected spring
	Bottled water
Improved: Sachet water	Sachet water
Unimproved: Surface water	Rain water
	Unprotected spring
	River, stream
	Dugout, pond, lake, dam, canal
Unimproved: Others	Tanker supply, vendor provided
	Unprotected well
	Other

* The GLSS surveys further divide pipe-borne (outside dwelling) into cases where: (i) the pipe is not on the compound, and (ii) a neighbour’s pipe is used.

To identify temporal trends in the consumption of different water sources we counted the number of households identifying each source as their primary water source at three administrative levels (country, region, district). Within these levels we also studied the variation between rural and urban households, and households found in Greater Accra. To identify the extent of spatial clustering in sachet water consumption we computed the global Moran’s I at the district level for each time point in the study period. We then calculated the local Moran’s I to show how spatial autocorrelation varied across the country. These results were interpreted by deriving the Local Indicators of Spatial Association (LISA) clusters for the study region [38].

#### National and subnational development trends

In the next part of the analysis we focused on Ghana’s development trends during the study period. Living standards were estimated through the identification of relevant household characteristics which were consistently recorded across the three surveys. The indicators relate to the dwelling itself (type, ownership), access to electricity (source of lighting), access to sanitation services (type of toilet, liquid and solid waste disposal methods, type of water used for non-drinking purposes), type of cooking fuel, and access to technology (mobile phone, computer). For each indicator, the possible answers were aggregated to opposing pairs which generally represented positive and negative categories (with the exception of the type of toilet, where we used three categories to distinguish between water closets, Kumasi Ventilated Improved Pit (KVIP) latrine, and other types). Where indicators referred to accessibility to a service the derivation of categories was for the most part self-explanatory (i.e. has access/does not have access). We divided cooking fuels into solid and liquid types, reflecting the fact that the use of solid fuels tends to be associated with higher levels of household poverty [39]. However, we note that the energy stacking hypothesis calls in to question this assertion, and therefore urge caution in the interpretation of these results [40]. All the selected indicators have been used previously as proxies of living standards [23, 39, 41–43, e.g.]. Once these categories were defined we computed the percentage of households exhibiting the different aspects of each indicator at the district level.

We supplemented the development indicators with the Subnational Human Development Index (SHDI) [44] to gain a broader understanding of Ghana’s development trends beyond the household characteristics outlined above. The SHDI is a metric based on the United Nations’ Human Development Index (HDI), a composite index of life expectancy, education and per capita income available at the country level. The SHDI extends the HDI methodology with additional publicly available data to produce analogous data at a finer geographical level. For Ghana the SHDI data is available for the 10 administrative regions used in the analysis (Fig 1). Other than the SHDI itself we also considered some of its constituents, namely the gross national income (GNI), education index (EI) and health index (HI).

Trends in development from both the census data and the SHDI were regressed against trends in changing drinking water sources over the study period using repeated measures correlation [45]. This was achieved by considering the regional (*n* = 10) data in 2010, 2013, and 2017 as separate data points, giving a total of 30 data points on which to perform the regression.

## Results

**Fig 2:**
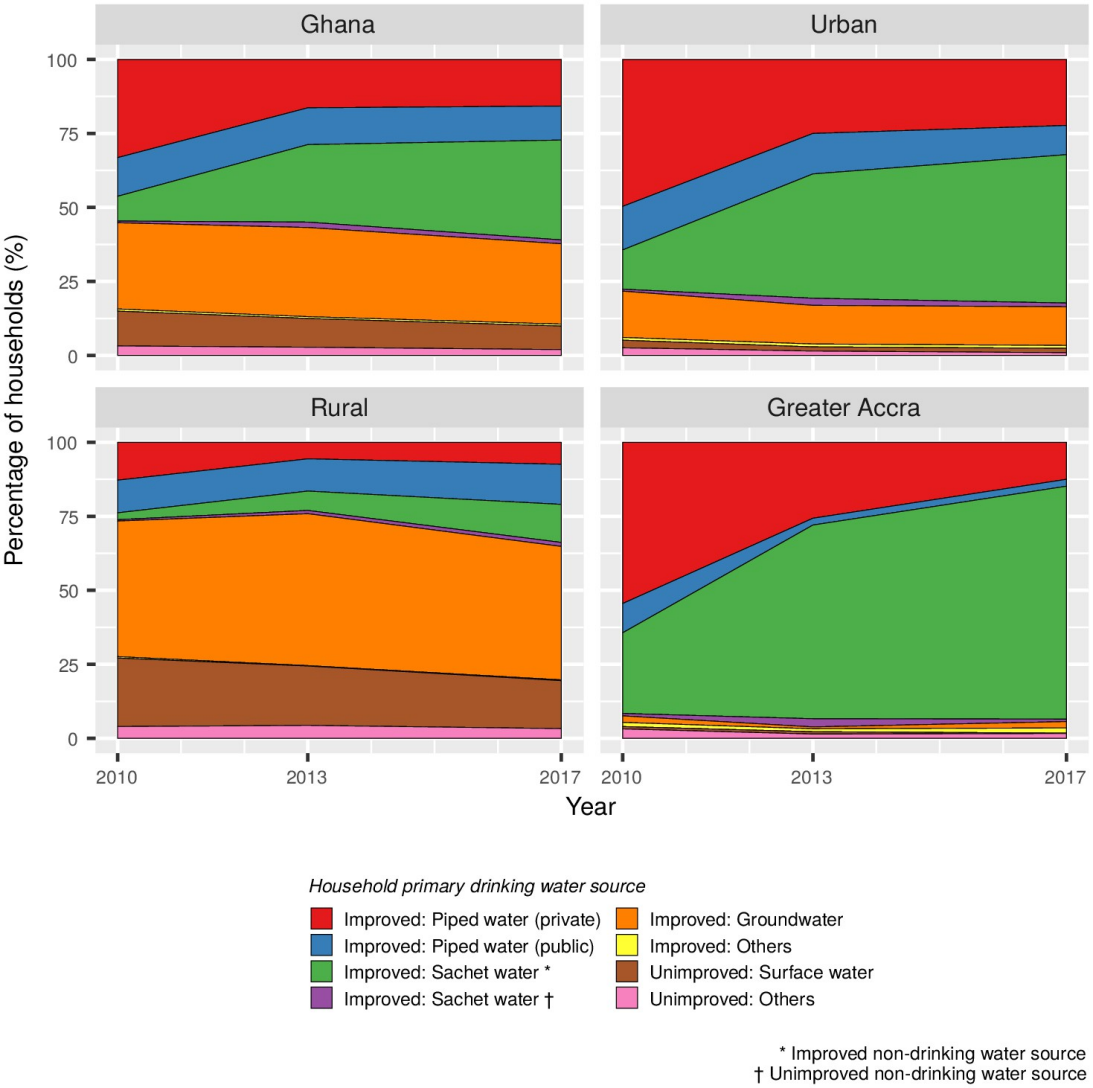
Primary drinking water source for households in Ghana as a percentage of the total number of households.

The number of Ghanaian households identifying sachet water as their preferred source of drinking water increased fivefold between 2010 and 2017 (Fig 2). By the end of the study period in 2017, sachet water was consumed by 35% of households as their primary source of drinking water, mostly driven by increasing consumption in urban areas. The consumption of piped water decreased from 46% of households in 2010 to 27% in 2017, corresponding to a fall of around 750,000 households in absolute terms (S1 Fig). In 2017 the vast majority of households in Ghana consumed water from an improved source, increasing from 85% to 90% between 2010 and 2017. By 2017, the number of households drinking unimproved water sources had decreased by around 165,000 from a total of almost 900,000 in 2010.

In urban areas the consumption of sachet water has risen both in terms of the total number of households and market share. Between 2010 and 2017, the number of urban households reporting sachet water as their preferred drinking water source increased from 450,000 to 2.1 million (S2 Fig). The growth in sachet water consumption coincides with an increase of around 810,000 households during the study period, as well as a fall in the number of households consuming piped water of approximately 800,000, from a total of 2.11 million in 2010. The number of urban households consuming groundwater increased slightly by about 20,000 to a total of 535,000 households in 2017. By the end of the study period in 2017 sachet water was the preferred drinking water source of 51% of urban households, compared to 14% in 2010.

There was a sixfold increase in the number of rural households consuming sachet water as their preferred drinking water source, from around 70,000 households in 2010 to 450,000 households by 2017, representing a market share of 14% (S2 Fig). The number of households reporting unimproved surface water as their main drinking water source decreased from approximately 720,000 in 2010 to 630,000 in 2017, despite an increase in the number of rural households of around 560,000 over the study period.

In Greater Accra, sachet water was the primary source of drinking water for 80% of households in 2017. Between 2010 and 2017, the market share of sachet water increased from 28% to 80% of households, representing a threefold increase from 310,000 to 1.05 million households identifying sachet water as their primary water source during the study period (S3 Fig). The consumption rate of sachet water in Greater Accra was higher than the national average across all the years considered.

**Fig 3:**
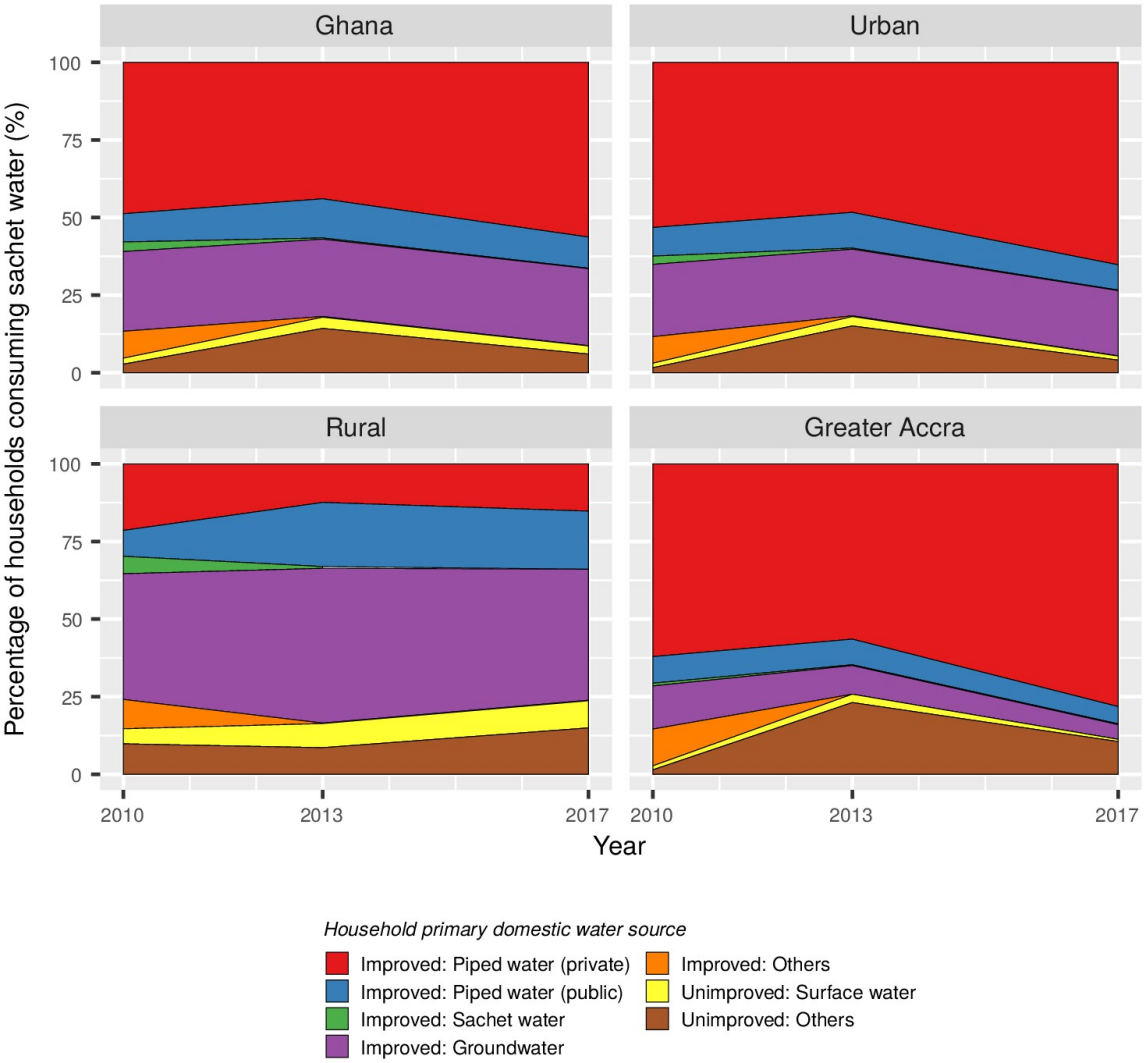
Primary water source for domestic uses amongst households citing sachet water as their primary source of drinking water.

In 2017 more than 90% of households using sachet water as their main source of drinking water used an improved water source for their household uses (Fig 3). In urban areas the percentage of sachet consumers with a private connection to the piped supply network increased from around 53% in 2010 to 65% in 2017, while in Greater Accra the percentage increased from 62% to 78%. Nevertheless, in 2017 around 10% of households consuming sachet water in Greater Accra relied on unimproved sources for their household uses. In rural areas the majority of sachet water consumers used groundwater for household uses, although 23% of households relied on an unimproved source.

**Fig 4:**
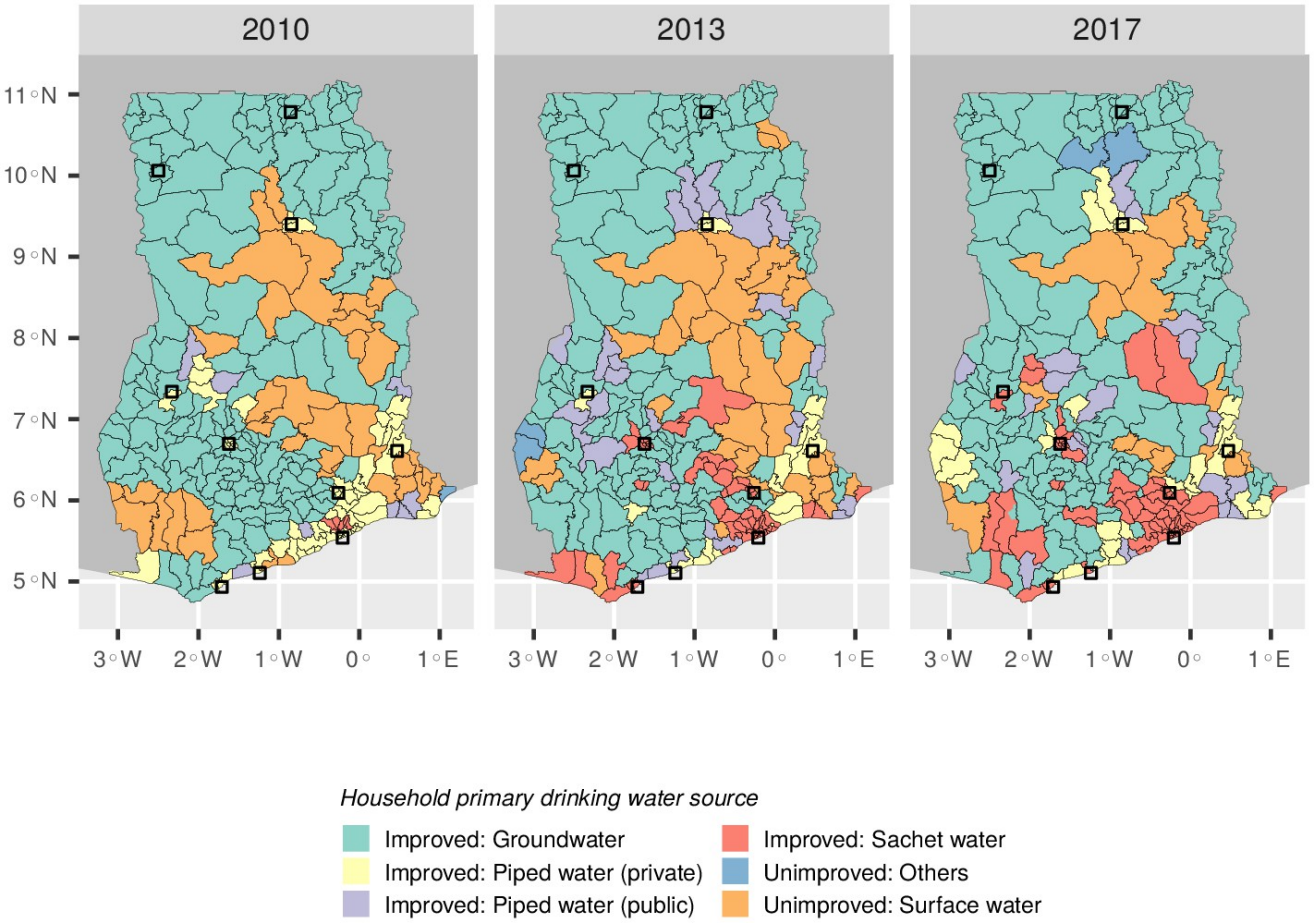
Most common source of drinking water in Ghana’s 170 districts. Maps were created with data from the Humanitarian Data Exchange [33], which are made available under a CC-BY license [34]. For copyright reasons the district boundaries shown are from the present day (*n* = 260).

**Fig 5:**
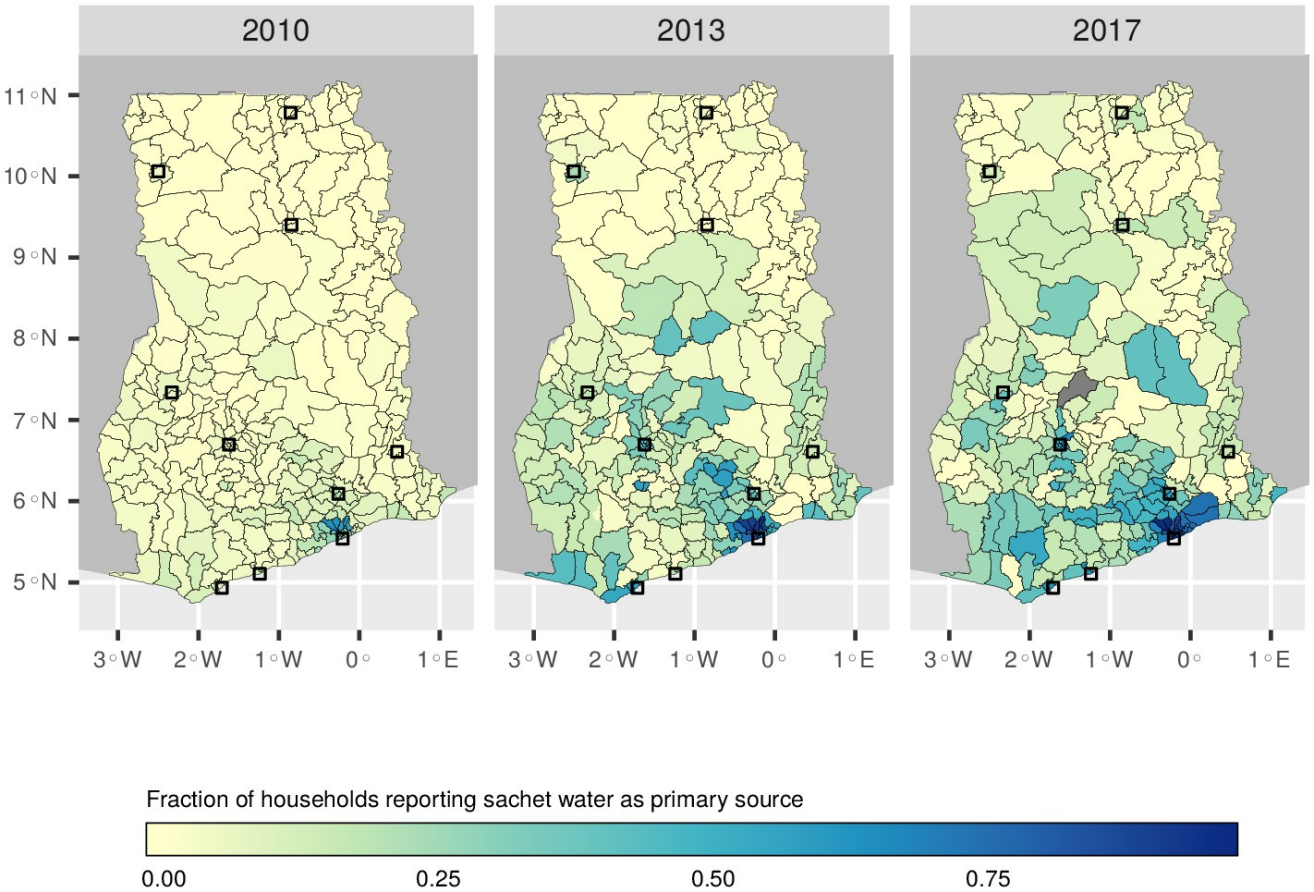
Percentage of households drinking sachet water in Ghana’s 170 districts. Maps were created with data from the Humanitarian Data Exchange [33], which are made available under a CC-BY license [34]. For copyright reasons the district boundaries shown are from the present day (*n* = 260).

The number of districts (*n* = 170) where sachet water is the most common drinking water source increased from three to 36 between 2010 and 2017 (Fig 4). Groundwater was the most common in 88 districts—the highest number of any drinking water source—mainly due to its predominance in rural regions. In general, districts consuming sachet water tend to cluster in the southern half of the country (Fig 5). Here, sachet water consumption appears to have grown fastest in the regions surrounding Accra, spreading to other coastal regions before moving inland towards Kumasi.

**Fig 6:**
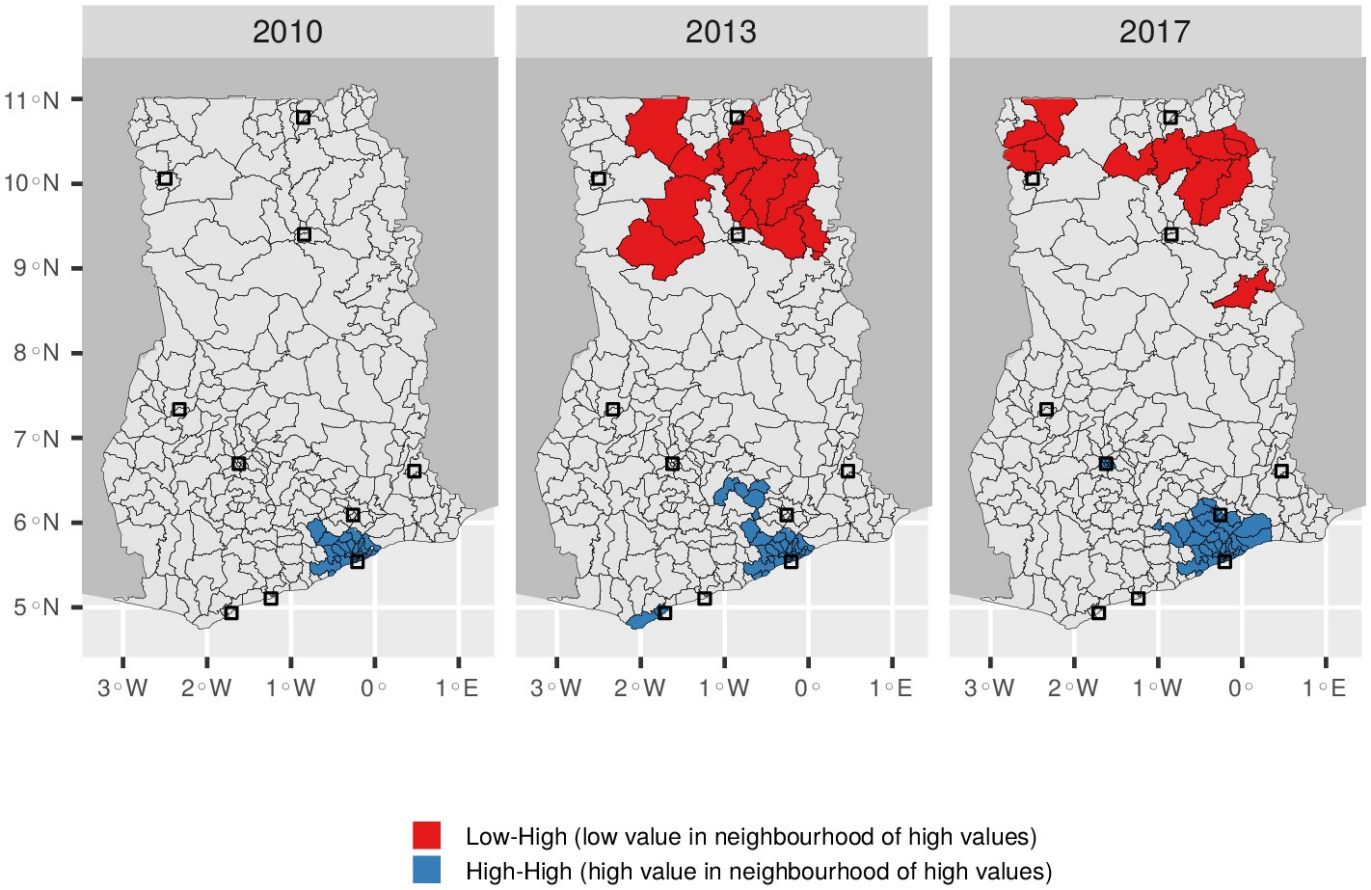
Local Indicators of Spatial Association clusters for Ghana in 2010, 2013 and 2017. Maps were created with data from the Humanitarian Data Exchange [33], which are made available under a CC-BY license [34]. For copyright reasons the district boundaries shown are from the present day (*n* = 260).

Spatial autocorrelation is observed in all years (Table 2), suggesting that districts with a relatively high rate of sachet water consumption tend to be clustered. The degree of clustering increased slightly between the years considered, suggesting that sachet water could be a phenomenon which spreads from high consumption districts to neighbouring low consumption districts as user habits and manufacturing expertise cross district boundaries. The LISA analysis reveals that clustering has tended to occur in the southern part of the country, notably around Accra in all years but most recently around Kumasi (Fig 6).

**Table 2:** Spatial autocorrelation between sachet consumption and household density in 2010, 2013 and 2017.

Year	Moran’s I	p-value	z-score
2010	0.59	<0.001	13.17
2013	0.59	<0.001	12.49
2017	0.62	<0.001	12.84

Moran’s I measures spatial autocorrelation based on the location and values of a set of features, bounded by the interval [-1, 1]. Values approaching the upper and lower bounds indicate strong clustering and dispersion, respectively, while values near zero suggest a random spatial distribution.

Indicators of improved living conditions have generally become more prevalent between 2010 and 2017 (Table 3), including access to electrical lighting, improved toilets (WC or KVIP), improved cooking fuel (gas, electric or kerosene), waste collection service, connection to a sewage network, and access to computers and mobile phones. Home ownership saw a consistent decrease between 2010 and 2017. The increase in living standards shown by the indicators in Table 3 is consistent with the changes in the SHDI and its constituents during the study period.

**Table 3:** Distribution of household characteristics in 2010, 2013 and 2017.

Characteristic	Rural			Urban (excl. Accra)			Urban (Accra only)		
	2010	2013	2017	2010	2013	2017	2010	2013	2017
Type of dwelling									
House, dwelling, flat	46.3	25.8	42.2	34.3	24.4	30.6	30.5	28.3	32.3
Compound, hut, tent, kiosk, business, other	53.7	74.2	57.8	65.7	75.6	69.4	69.5	71.7	67.7
Source of lighting									
Electric	40.1	48.6	70.5	82.0	86.6	91.6	89.6	93.0	94.8
Gas, wood, other	59.9	51.4	29.5	18.0	13.4	8.4	10.4	7.0	5.2
Type of toilet									
WC	3.3	2.3	4.5	21.2	18.3	25.0	32.3	33.5	37.0
KVIP	7.5	8.2	11.9	12.2	12.8	15.1	14.4	20.5	16.1
Pit latrine, bucket, other, none	89.2	89.5	83.7	66.6	68.9	59.9	53.3	46.0	46.9
Type of cooking fuel									
Gas, electricity or kerosene	5.4	5.7	8.9	23.7	28.5	30.0	44.9	53.3	55.1
Wood, other	94.6	94.3	91.1	76.3	71.5	70.0	55.1	46.7	44.9
Solid waste disposal method									
Collection service	4.6	3.8	3.7	8.9	12.6	22.3	50.8	66.0	69.3
Dump, Burnt, buried, dumped	95.4	96.2	96.3	91.1	87.4	77.7	49.2	34.0	30.7
Liquid waste disposal method									
Sewage network	5.8	5.9	4.5	22.3	32.1	38.0	34.4	54.4	59.7
Other	94.2	94.1	95.5	77.7	67.9	62.0	65.6	45.6	40.3
Access to computer (desktop or laptop)									
Has access	2.2	4.3	7.4	10.0	16.6	18.9	17.3	29.0	28.8
Does not have access	97.8	95.7	92.6	90.0	83.4	81.1	82.7	71.0	71.2
Access to mobile phone									
Has access	55.9	77.7	87.7	83.7	92.6	96.8	92.8	96.0	97.8
Does not have access	44.1	22.3	12.3	16.3	7.4	3.2	7.2	4.0	2.2
Type of water used for non-drinking purposes									
Improved	70.3	70.1	76.7	93.3	91.9	94.5	98.6	97.1	99.1
Unimproved	29.7	29.9	23.3	6.7	8.1	5.5	1.4	2.9	0.9
Ownership of dwelling									
Owned by household	65.4	62.1	57.9	33.7	31.6	30.5	30.7	35.0	27.8
Renting, rent-free, perching, other	34.6	37.9	42.1	66.3	68.4	69.5	69.3	65.0	72.2
Gender of household head									
Male	69.2	74.0	69.2	61.2	64.4	63.4	64.3	68.7	67.4
Female	30.8	26.0	30.8	38.8	35.6	36.6	35.7	31.3	32.6

**Fig 7:**
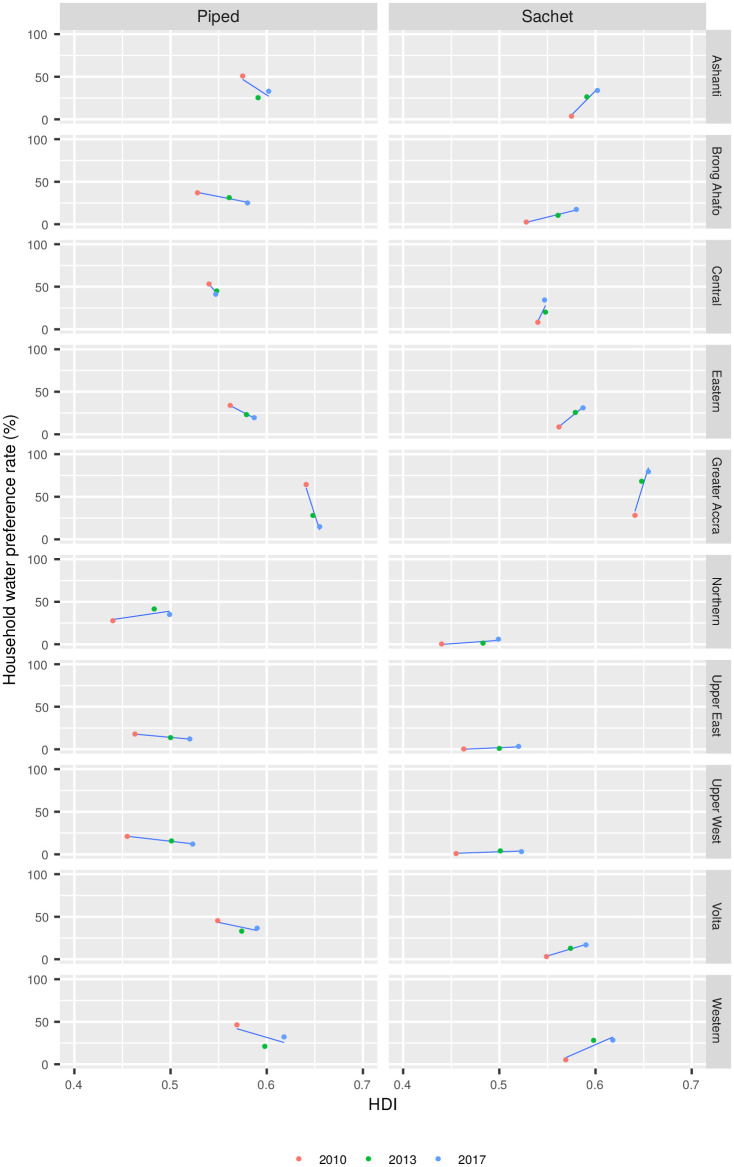
Correlations between the SHDI and the usage of sachet water or piped water as primary drinking water sources.

Sachet water consumption is positively correlated with multiple indicators of improved living conditions (Table 4). It is strongly correlated with the use of gas, electricity or kerosene as cooking fuel in both rural (r = 0.86, p<0.001) and urban (r = 0.79, p<0.001) contexts. Possession of a computer is also correlated with sachet water consumption in both urban and rural settings. Among household characteristics related to sanitation, there is a strong correlation between sachet water consumption in urban areas and access to the sewage network (r = 0.75, p<0.001). Sachet water consumption is also positively correlated with SHDI and its constituents. For the most part, we find that an increase in SHDI has corresponded with an increase in the consumption of sachet water and a decrease in the consumption of piped water for drinking water (Fig 7).

**Table 4:** Correlation between household characteristics and consumption of water types at the regional level. Only values significant at the 5% level are reported.

Characteristic	Rural				Urban			
	Sachet	p-value	Piped	p-value	Sachet	p-value	Piped	p-value
Lighting: Electric	0.60	0.004			0.56	0.008	-0.44	0.044
Lighting: Gas, wood, other	-0.60	0.004			-0.56	0.008	0.44	0.044
Toilet: WC	0.70	<0.001	-0.51	0.017				
Toilet: KVIP							-0.56	0.008
Toilet: Pit latrine, bucket, other, none	-0.63	0.002			-0.46	0.037	0.57	0.006
Cooking fuel: Gas, electricity or kerosene	0.86	<0.001	-0.68	<0.001	0.79	<0.001	-0.75	<0.001
Cooking fuel: Wood, other	-0.86	<0.001	0.68	<0.001	-0.79	<0.001	0.75	<0.001
Solid waste: Collection service					0.65	0.001	-0.62	0.003
Solid waste: Dump, Burnt, buried, dumped					-0.65	0.001	0.62	0.003
Liquid waste: Sewage network					0.75	<0.001	-0.62	0.003
Liquid waste: Other					-0.75	<0.001	0.62	0.003
Technology: Has computer	0.86	<0.001	-0.50	0.020	0.88	<0.001	-0.74	<0.001
Technology: Does not have computer	-0.86	<0.001	0.50	0.020	-0.88	<0.001	0.74	<0.001
Technology: Has mobile phone	0.45	0.040			0.70	<0.001	-0.57	0.007
Technology: Does not have mobile phone	-0.45	0.040			-0.70	<0.001	0.57	0.007
Non-drinking water: Improved	0.48	0.029						
Non-drinking water: Unimproved	-0.48	0.029						
Home ownership: Owned by household	-0.65	0.001						
Home ownership: Renting, rent-free, perching, other	0.65	0.001						
Household head: Female					-0.46	0.036		
Subnational HDI					0.59	0.005	-0.55	0.010
Gross National Income	0.66	0.001			0.92	<0.001	-0.84	<0.001
Health Index	0.48	0.029			0.60	0.004	-0.61	0.004

Note that housing characteristics (*House, dwelling, flat* versus *Compound, hut, tent, kiosk, business, other*) and *Education Index* showed no significant correlation with sachet water consumption in either rural or urban areas, and have therefore been omitted from the table.

The rate of sachet water consumption varies by geography and household characteristics (Tables 5 and 6). Between 2010 and 2017 the rate of sachet water consumption increased in households with and without characteristics indicative of improved living conditions, suggesting that sachet water is a phenomenon gaining popularity in all sections of society. Households with indicators of better living conditions have taken up sachet water at higher rates. The consumption of sachet water among households with WC or KVIP as their toilet type increased more than households using less sophisticated means such as pit latrines or buckets. Similar patterns were observed in waste disposal characteristics, where urban households with access to the sewage network or rubbish collection services saw higher uptakes of sachet water in comparison to households with less sophisticated methods of waste disposal.

**Table 5:** Sachet water use among households of different characteristics.

Characteristic	Rural			Urban (excl. Accra)			Urban (Accra only)		
	2010	2013	2017	2010	2013	2017	2010	2013	2017
Type of dwelling									
House, dwelling, flat	2.5	10.0	13.7	8.5	36.6	44.7	29.0	73.3	71.5
Compound, hut, tent, kiosk, business, other	3.0	6.8	14.6	7.1	30.8	37.2	27.6	68.8	83.7
Source of lighting									
Electric	5.7	13.6	18.5	8.3	35.1	41.3	28.3	70.6	80.2
Gas, wood, other	0.8	2.0	3.9	4.0	13.4	19.2	25.3	63.7	71.2
Type of toilet									
WC	17.6	38.4	50.1	11.0	48.5	56.6	30.5	70.7	79.4
KVIP	6.5	10.8	21.1	10.8	37.1	43.4	31.1	62.2	82.6
Pit latrine, bucket, other, none	1.9	6.6	11.3	5.8	26.9	31.3	25.7	73.1	79.1
Type of cooking fuel									
Gas, electricity or kerosene	20.2	42.9	60.3	14.3	51.5	61.7	32.1	73.0	81.5
Wood, other	1.8	5.5	9.7	5.5	24.5	29.9	24.6	66.8	77.6
Solid waste disposal method									
Collection service	9.2	14.9	41.1	10.7	39.2	57.1	30.4	72.1	81.0
Dump, Burnt, buried, dumped	2.5	7.3	13.2	7.2	31.2	34.4	25.5	66.2	76.9
Liquid waste disposal method									
Sewage network	6.3	21.7	34.8	7.0	45.1	48.9	25.2	68.8	83.8
Other	2.6	6.8	13.2	7.7	26.1	33.7	29.5	71.6	73.7
Access to computer (desktop or laptop)									
Has access	19.1	33.1	36.6	15.0	49.1	54.7	34.1	76.1	76.9
Does not have access	2.4	6.5	12.4	6.7	28.8	35.9	26.7	67.7	80.9
Access to mobile phone									
Has access	4.5	9.0	15.7	8.5	33.7	40.3	28.4	70.4	79.7
Does not have access	0.6	2.9	3.4	3.0	12.7	13.2	22.4	62.0	82.9
Type of water used for non-drinking purposes									
Improved	3.3	9.3	16.8	7.4	32.5	40.2	27.7	69.5	79.8
Unimproved	1.6	3.7	5.8	10.0	29.1	27.6	46.3	88.7	72.3
Ownership of dwelling									
Owned by household	1.4	4.2	8.5	5.3	24.0	27.5	24.9	63.4	70.2
Renting, rent-free, perching, other	5.4	13.3	22.1	8.7	36.0	44.7	29.4	73.7	83.4
Gender of household head									
Male	2.6	7.0	14.0	8.1	34.1	41.0	28.5	69.9	78.7
Female	3.1	9.4	14.8	6.7	28.7	36.9	27.2	70.5	81.9

**Table 6:** Sachet water use among households by region.

Region	Rural			Urban		
	2010	2013	2017	2010	2013	2017
Ghana	2.8	7.6	14.2	13.9	44.4	51.4
Greater Accra	28.8	37.3	76.7	28.0	70.1	79.8
Ashanti	1.1	8.6	15.2	5.1	39.9	45.0
Brong Ahafo	0.7	3.8	7.5	4.8	16.6	28.2
Central	3.2	5.5	19.5	13.7	36.5	51.5
Eastern	3.8	14.8	17.4	14.0	37.1	46.9
Northern	0.3	0.3	2.7	0.6	3.1	10.7
Upper East	0.2	0.1	2.0	0.8	4.2	7.9
Upper West	0.3	0.8	1.0	3.0	16.0	12.2
Volta	1.3	4.6	8.0	6.8	28.8	32.1
Western	3.1	13.0	15.6	8.2	47.1	43.1

## Discussion

Despite its origins as a niche product in Accra, sachet water is now consumed throughout Ghana. In general it remains an urban phenomenon, although it is increasingly used by rural households. Consumption continues to be highest in Accra, where sachet water first gained a foothold in Ghana’s water market. There is evidence that districts with a high incidence of sachet water consumption are clustered, suggesting that the market for sachet water may expand from urban centres. This is most evident in districts surrounding Accra, in which the percentage of households citing sachet water as their main drinking water source has increased from less than 20% in 2010 to more than 70% by 2017. Usage appears to have spread westwards from Accra along the coast and inland towards Kumasi, Ghana’s second largest city (Fig 5). While previous research showed that households consuming sachet water as their primary drinking water source tended to be from socioeconomically disadvantaged backgrounds [10, 22], our results show that sachet water is increasingly consumed by housholds with indicators of relative wealth. This shows that the market has changed significantly since 2010, when it was associated mainly with the “poorest of the poor” [10], and points to the challenges facing Ghana’s water utility as it seeks to keep pace with the country’s economic development. Accordingly, we find that sachet water is now a ubiquitous product in Ghana, consumed across socioeconomic groups, which is playing an increasingly important role in the provision of drinking water to its population.

Our analysis shows that many urban households are choosing sachet water as their primary source of drinking water despite having access to another improved water source (Fig 3). This is most apparent in Greater Accra, where 78% of households used sachet water as their primary drinking water source despite having a private connection to the piped supply network. However, although piped water is nominally considered an improved source under the JMP, the perceived and actual quality of water from Ghana’s piped system is highly variable [5]. In Accra, the relative quality of sachet water is likely to be the main driver of the shift away from piped water [3, 23]. Here, the notion of sachet water being of superior quality to piped water is well established and regularly exploited by vendors of sachet water [46]. This is not without reason: Accra’s decaying pipe network is made up of shallow, occasionally exposed pipes which often run adjacent to sewers [5]. The reliability of piped water is also highly variable [2]. For instance, water rationing is used in Accra to manage the deficit between supply and demand, with some residents going up to a week without receiving piped water [15]. The relatively low unit price of sachet water, which bridges the large gap between bottled water and piped water [3], enables the industry to exploit the inadequate municipal supply.

With urbanisation expected to continue unabated across sub-Saharan Africa for at least the next three decades [47], it is unlikely that Ghana will have the capacity to provide universal access to its piped supply network [5, 46]. The potential contribution of sachet water towards SDG 6 is obvious, with many commentators suggesting that sachet water is likely to play an important role in achieving universal access to improved drinking water. As Stoler [3] states, “[t]he ability of many West African nations to achieve universal access to safe drinking water may depend on their willingness to understand and incorporate the sachet water industry into an integrated drinking water platform.” Williams *et al*. [13] agrees, arguing that “policymakers and regulators should recognize the potential benefits of packaged water in providing safer water for consumption at and away from home, especially for those who are otherwise unlikely to gain access to a reliable, safe water supply in the near future.” The ubiquity of sachet water in Accra, and its growing presence elsewhere in the country, suggests that sachet water is already an indispensable part of Ghana’s water landscape.

There is broad agreement about the need for better governance of the packaged water industry to ensure it supports the drive towards universal access without compromising other aspects of Ghana’s development agenda. Important issues discussed in the literature include how best to achieve consistent water quality standards, as well as how to curb the environmental impact associated with the production of vast quantities of single-use plastic waste [5]. With respect to water quality, Stoler *et al*. suggest that in areas where the sachet market is mature and competitive the quality of water generally exceeds drinking water standards [3, 22], although there remains considerable variability amongst smaller producers [48]. Outside of large urban centres the quality is less assured due to the predominance of smaller manufacturers [9, 12, 13]. There are also outstanding questions about the potential impact of plastic degradation on water quality [3]. On the environmental impact, plastic waste associated with sachet water consumption has been linked to blocked drains, localised flooding, and ecological degradation [49]. Wardrop *et al*. [50] estimated that in 2015 around 8.2 billion sachets were consumed, producing approximately 14,000 tonnes of plastic waste. Outright bans on plastic sachets are periodically suggested as a way to curb the amount of waste which is generated by the sachet water market [3, 49]. However, inadequate solid waste management in Ghana is a problem which transcends any particular sector or consumer product, and instead speaks to the inability of municipal authorities to provide sufficient solid waste disposal services [51, 52]. Indeed, addressing the lack of solid waste management in Ghana is a critical development challenge which intersects many of the SDGs [53].

Aside from the practical considerations of water quality and environmental degradation, there are concerns that Ghana’s rapidly expanding sachet water market may threaten the ideals of universal access to affordable drinking water [5]. Left unchecked, sachet water has the potential to directly compete with the public water utility [6], undermining the traditional notion of water as a basic human right and establishing a system of water governance which views water as a discrete private good [54]. In Ghana the provision of sachet water is not viewed by the state as part of an essential public service [5], even though for many Ghanaians it is their only source of clean drinking water [55]. On the one hand, the lack of regulation leaves consumers vulnerable to price fluctuations, varying water quality, and inconsistent supply [3, 7, 54]. Despite some efforts towards regulating water quality, including the introduction of a quality seal issued to producers who pass an inspection by Ghana’s Food and Drug Authority [56], the sheer number of producers—many of which are unregistered [5]—renders the enforcement of regulations a Sisyphean task. On the other hand, it is true that considerable progress on water quality and price stability have been achieved through competition and industrialisation alone. While Ghana’s overall committment to universal access to water is assured, the means by which this is achieved requires careful monitoring to ensure that the most vulnerable members of society are not further disadvantaged by the increasing reliance on packaged water.

As the sachet water industry spreads across the country to participate in diverse water markets it appears increasingly unlikely that a top-down regulatory framework will be effective at addressing quality and environmental concerns and widening access to clean, affordable drinking water [54, 57]. In the face of rapid environmental and socioeconomic change the involvement of local stakeholders in managing their water is recognised as a key strategy for achieving water security, as advocated by SDG 6.b (“support and strengthen the participation of local communities in improving water and sanitation management”) [25]. It therefore seems likely that integrating the sachet water market into participatory water governance mechanisms offers the best chance for reestablishing the principle of water as public service [13, 54]. Such efforts could perhaps be modelled on the Local Water Boards which currently operate in Accra, even though to date these have faced considerable challenges in maintaining access to affordable drinking water [55, 58]. Indeed, there is a need for further empirical research towards effective water governance in Africa’s rapidly expanding cities and towns [3]. On this point, a critical analysis of Ghana’s initiative to provide free water for all during the Covid-19 pandemic may provide valuable evidence on the ability of the state to harness sachet water to guarantee the supply of clean drinking water to its constituents [59].

Nevertheless, participatory approaches to water governance should not be seen as a panacea, nor absolve the state of its ultimate responsbility to provide its citizens with universal access to safe and affordable drinking water. Widespread public distrust of tap water is clearly a major driver of the observed trend in sachet water consumption, especially amongst households which have access to another improved water source [15]. Renewing confidence in the quality of tap water should therefore be a matter of priority for the water utility, backed up by remedial works where necessary. Lastly, we highlight that the relative cost of sachet water means that it can only realistically alleviate the demand for clean drinking water [5]. The focus on sachet water as a solution to drinking water tends to overshadow the shortage of water for other domestic uses, including sanitation [5, 60]. This is part of a wider development trend which has tended to promote efforts aimed at improving drinking water access over sanitation needs [35]. According to the government’s own estimates only 15% of Ghana’s population have access to improved sanitation facilities, with the state of sanitation especially poor in urban areas [61]. While the ubiquity of sachet water may reduce the level of dependence on the piped network for meeting drinking water demand, the extension of the public water supply is likely to be a necessary feature of efforts to raise sanitation standards and enhance the water security of Ghana’s urban residents.

### Conclusion and future outlook

We studied three nationally representative datasets from 2010, 2013 and 2017 to better understand the changing demography of sachet water consumers in Ghana. The results show that sachet water is now a ubiquitous source of drinking water in Ghana which is used in both urban and rural settings. The capital city, Accra, remains the largest consumer of sachet water. However, during the study period sachet water consumption has spread westwards along the coast and inland to Kumasi, Ghana’s second city. Despite a 34% increase in Ghana’s population between 2010 and 2017, the percentage of households with access to improved water increased, due in part to the growth in sachet water usage.

Households with indicators of relative wealth increased their consumption of sachet water during the study period. In 2017, the majority of households citing sachet water as their primary source of drinking water already had access to an improved water source. On the one hand, this suggests that for some households the switch to sachet water from piped water has been by choice rather than necessity. It is possible that increasing prosperity has enabled some households to meet their drinking water demand with sachet water because of pre-existing concerns (real or perceived) about the quality and reliability of the piped supply network. That said, it should be noted that we did not attempt to establish causal relationships between variables. Although concerns about the quality and environmental impact of sachet water abound, additional regulation of the industry is fraught with difficulty. In the face of climate change, socioeconomic development, and rapid urbanisation, we suggest that strengthening participatory mechanisms for water governance is a practicable way forward to ensure that Ghana’s growing sachet water industry makes a positive contribution towards SDG 6.

The survey datasets we have analysed have some important characteristics and shortcomings which should be taken into account when interpreting our results. Most importantly, we reiterate that the datasets inquired about water preferences at the household level and not the individual level. It is likely that different members of the the household have different preferences for water depending on their daily lives and routines. Moreover, previous research has observed that households in Ghana tend to have multiple sources of drinking water [9, 21]. While the censuses capture relatively fine-grained information on the water sources available to households, they do not provide insight into the motivations behind the choice of one drinking water source over another. We suggest the question asked by Stoler *et al*. [23]—“why do you use or not use sachet water?”—could be used as a reference in the design of future large-scale surveys to gain additional information about consumer choices. In our analysis we used the UNICEF/WHO JMP definitions to classify drinking water sources as improved or unimproved. However, due to limitations of the survey data it was not possible to apply the complete JMP service ladder [37]. Further work should consider how the service ladder could be applied to Ghanaian census data, as this may provide valuable insights into the drivers of sachet water consumption.

It is important to bear in mind that the indicators of living standards we have used in our analysis represent those which could be applied with some confidence across urban and rural households nationally. The indicators do not encompass every potential source of improved living standards, and it is inevitable that we may have disregarded some indicators which are important only in certain regions or urban/rural contexts. Our analysis in the spatial dimension was constrained by the relatively coarse resolution of the GLSS datasets, in which data points are georeferenced to the district level (*n* = 170). As a result, our analysis inevitably fails to pick up on important spatial heterogeneities which may provide additional insight into the physical and socioeconomic drivers of sachet water consumption. In contrast, the Population and Housing Census—which surveys the entire population—georeferences responses to enumeration areas (*n* = 37, 642). In this respect, the results of the forthcoming Population and Housing Census, postponed from 2020 to 2021 as a result of the Covid-19 pandemic, will likely be highly relevant to the study of Ghana’s sachet water phenomenon.

## Supporting information

S1 Fig Primary drinking water source for households in Ghana. (PDF)

S2 Fig Primary drinking water source for urban and rural households. (PDF)

S3 Fig Primary drinking water source for households in Greater Accra. (PDF)
