# Supplementary material for: Sachet water in Ghana: A spatiotemporal analysis of the recent upward trend in consumption and its relationship with changing household characteristics, 2010–2017
Source: PLoS One. 2022 May 26;17(5):e0265167. doi: 10.1371/journal.pone.0265167 (PMC9135223; doi:10.1371/journal.pone.0265167)
Supplement: S1 Fig — (PDF) [file pone.0265167.s001.pdf]

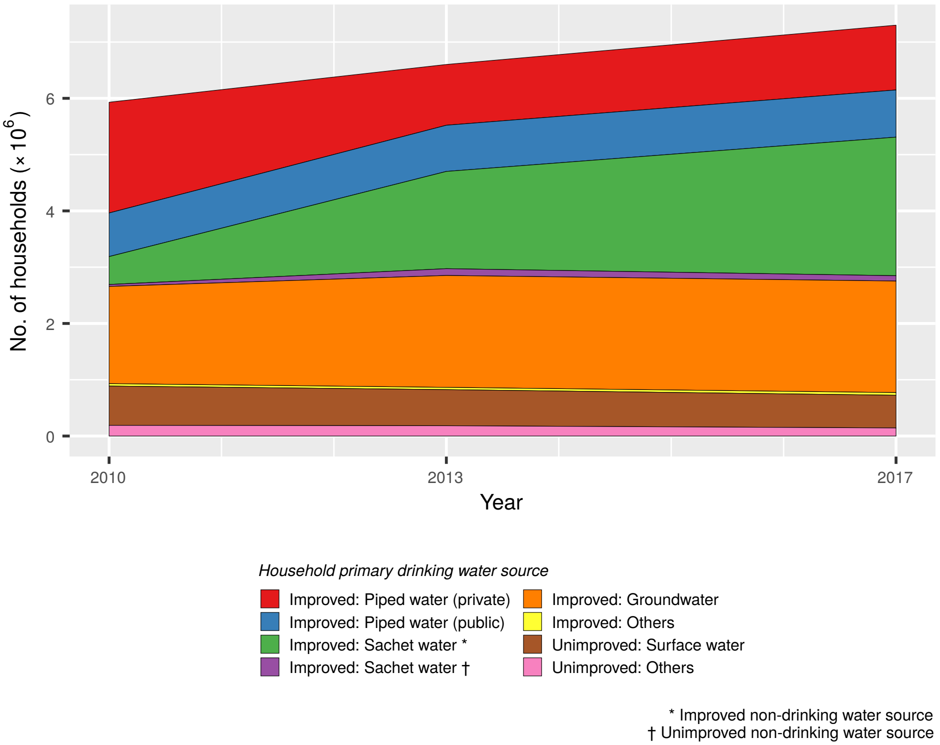

No. of households (×10^6)
0
2
4
6
2010
2013
2017
Year
Household primary drinking water source
Improved: Piped water (private)
Improved: Piped water (public)
Improved: Sachet water *
Improved: Sachet water †
Improved: Groundwater
Improved: Others
Unimproved: Surface water
Unimproved: Others
* Improved non-drinking water source
† Unimproved non-drinking water source
